# ‘Stay Curious’: guest experiences of philosophical counselling in palliative care – a qualitative study

**DOI:** 10.1186/s12904-026-02178-x

**Published:** 2026-06-29

**Authors:** Patrick Schuchter, Sandra Radinger, Stefanie Veronika Rieger, Klaus Wegleitner

**Affiliations:** 1 University of Applied Sciences Campus Vienna / Hochschule Campus Wien, Center for Applied Nursing Research / Zentrum für Angewandte Pflegeforschung, Favoritenstraße 222, Wien, A-1100 Austria; 2 Department of Linguistics, University of Vienna, Vienna, Austria; 3 Center for Interdisciplinary Research on Aging and Care (CIRAC), University of Graz, Graz, Austria; 4 Center for Interdisciplinary Research on Aging and Care (CIRAC), Institute of Pastoral Theology, University of Graz, Graz, Austria

**Keywords:** philosophical counselling, palliative care, end-of-life care, death literacy, existential inquiry, qualitative interviews, qualitative content analysis, communication

## Abstract

**Background:**

Philosophical counselling creates a dialogical space for exploring existential questions arising from serious illness, confrontation with mortality, bereavement, and caregiving. Although philosophy has long engaged with mortality and existential concerns, philosophy in form of philosophical counselling is not established in palliative care and has seldom been empirically studied. This study examines the views and experiences of individuals who engaged in philosophical counselling in palliative care–related contexts.

**Methods:**

Nine counselling processes were completed; post-counselling interviews were obtained from eight guests and analysed. Purposive sampling identified practitioners via professional associations in Austria, Germany, and Switzerland; guests were recruited by these practitioners in naturalistic practice settings. A qualitative content analysis was used to identify categories and subthemes.

**Results:**

Through iterative, interpretive analysis, we generated four main categories: distinctive features of philosophical counselling; how guests perceived the philosophical practitioner; perceived effects; and strengths and limits. Participants valued freedom to explore open-ended questions without pressure to act or solve problems. Conversations were described as inspiring and created space for a “stepping out”—being seen as a person rather than only as affected. We interpreted philosophical curiosity as a grounding stance toward mortality. At the same time, critical questioning could become destabilizing, underscoring the need for calibrated movement between personal involvement and abstract reflection within an empathetic dialogical relationship.

**Conclusions:**

Philosophical counselling seems to offer a space for reflective depth and existential inquiry in palliative care, fostering curiosity, wonder, and connectedness. It is described as offering intellectual freedom and an open, goal-free space. Practice implications include preserving this space, resisting help-driven agendas and didactic, overly abstract lecturing, and calibrating critical inquiry within empathetic dialogue. Philosophical counselling may contribute to facets of death literacy. Our findings indicate that end-of-life themes are already being addressed within philosophical counselling and offer guidance for introducing practice in palliative care contexts.

**Supplementary Information:**

The online version contains supplementary material available at https://doi.org/10.1186/s12904-026-02178-x.

## Background

Individuals facing severe illness, proximity to death, the loss of loved ones, or the need to care for others in such circumstances often encounter moments that give rise to fundamental questions about the key aspects of life. These questions frequently concern inherently philosophical themes such as the meaning and fulfilment of life, personal identity, guilt, freedom and responsibility, love and relationships, dignity, autonomy and connectedness, the diversity of ageing experiences, and the possibility of life after death [1–6].

We refer to these kinds of questions concerning the basic conditions and values of all human existence as “existential questions”. The idea of accepting dying and loss as valued parts of human experience has long been central to palliative care and the hospice movement. Moreover, addressing these existential questions is a core aspect of hospice- and palliative care [6–9]. Public health palliative care developments [10], which have become increasingly important over the last two decades, place particular emphasis on the social conditions and existential, spiritual, and cultural issues surrounding end-of-life care. This is evident, for example, in compassionate communities [11] initiatives or efforts to strengthen society’s competence in dealing with loss, dying, death, and grief— death literacy [12].

In philosophy, death and the existential questions associated with it have always been a central topic. According to Karl Jaspers [13], becoming aware of “ultimate situations”, such as facing death, is the origin of philosophical inquiry. Yet, philosophy as a way to encounter these existential questions has neither been systematically described nor empirically studied and is not an established part of palliative care.

The *Lancet Commission on the Value of Death* [14] emphasizes that a society’s approach to death is deeply embedded in philosophical traditions. It also argues that end-of-life care practices must take philosophical and spiritual dimensions seriously if we wish to reintegrate death into life and society. The Commission envisions a “realistic utopia” in which *“conversations and stories about everyday death*,* dying*,* and grief become common”* [14]. While initiatives such as Death Cafés or Last Aid courses [15] are suggested for realizing this vision, philosophical dialogues are conspicuously absent—despite the theoretical recognition of the philosophical dimensions of dying and grief. One way to make philosophical reflection relevant to everyday life is through “Philosophical Practice”. Philosophical Practice is today used as an umbrella term that includes: (1) philosophical counselling (one-on-one), (2) philosophizing with groups, and (3) broader public activities, like philosophical festivals [16]. In the German speaking area, Philosophical Practice is often considered a movement of re-connecting philosophy with everyday life outside universities and is traced back to Gerd Achenbach, who founded the first Philosophical Practice as institution for philosophical counselling in 1981 [17–20].

Three ideal-typical styles of philosophical counselling can be distinguished, though they often overlap in practice [1, 21–23]:

1. Hermeneutic-dialogical style: Guests share narrative experiences, and the philosophical conversation unfolds around an emergent “life-theme”. This approach, particularly common in German-speaking countries, often avoids strict methods (“no-method-style”) and views counselling as a creative art where the practitioner’s self-formation plays a key role [18, 19, 24].
2. Critical-thinking style: Focuses on clarifying concepts, logical analysis, and questioning assumptions. It often begins with a clear philosophical question and proceeds through systematic interrogation; this also includes Logic-Based Therapy (LBT) and Counselling as developed by E. D. Cohen, which systematises the identification of irrational premises and their refutation through philosophical reasoning [25–30].
3. Contemplative style: Integrates philosophical texts to open up reflective space. Guests reflect on their worldview and are invited to rethink their being-in-the-world through philosophical readings. Ran Lahav is a main representative of this style [31, 32].

Most literature on philosophical counselling is either (1) conceptual, exploring theoretical foundations [23, 31, 33] or drawing boundaries with psychotherapy [34, 35], or (2) anecdotal, with case studies by practitioners themselves [22, 24, 26, 36].

Philosophical practice in palliative care is mostly reflected in individual experiences, usually focusing on group formats [1, 37–39]. Philosophical *counselling* remains almost entirely unexamined as a central research subject. Notable exceptions include reflective accounts by Sesino [40], Diaz-Waian [41], Delgado [42], Lindseth [24], and a study based on self-observations by philosophical counsellors themselves in a hospital context [43].

Our research project “Philosophical Practice in Palliative Care and Hospice Work” addresses this gap on empirical insight into philosophical counselling. The project broadly asks: What does Philosophical Practice—currently or potentially—contribute to the development of a hospice-oriented culture of care in our society? What significance does it hold for end-of-life care? The research comprises several sub-studies, including an interview study with 29 philosophical practitioners, a focus group study, two participatory interventions—one with a mobile palliative care team and another within a regional, citizen-oriented Caring Community initiative—and, as presented in this paper, a study of nine individual counselling processes, each consisting of five sessions. These counselling processes were followed by interviews with the guests to gain insight into their view and experience of philosophical practice in palliative care and hospice contexts. We use ‘guest’ as the customary term for the counsellee/client in German-speaking philosophical practice, where this usage is widely spread. The following sub-question was at the heart of this project component: How do guests experience philosophical counselling?

## Methods

To gain insight into the guests’ experience and evaluations, we conducted, as part of a broader study, an exploratory qualitative study (interpretivist, moderately constructivist stance) using semi-structured interviews with eight guests of philosophical practitioners; data were analysed using qualitative content analysis with hybrid deductive (guide-derived) and inductive (open) coding, supported by ongoing team discussions.

### Data collection

In the individual counselling study, seven philosophical practitioners conducted nine counselling processes processes with nine guests, each consisting of five sessions, resulting in a total of 45 philosophical counselling sessions. These sessions were audio-recorded and subsequently transcribed. Of nine guests, eight completed post-counselling interviews included in this analysis. The analysis of the counselling conversations themselves is *not* the focus of the present paper.

Instead, we examined the interviews that were led with guests following the counselling processes. After the five sessions of philosophical counselling were completed, qualitative semi-structured interviews [44, 45] were conducted with eight guests by a member of the research team (researcher B). The interviews targeted four types of insights: (1) the guests’ experience of philosophical counselling, (2) their descriptions of philosophical counselling, (3) their evaluative statements about the experienced philosophical counselling, and (4) factual biographical information about the participant. Framed by an introductory and a closing phase ending in factual questions for (4), (1) and (2) were generated in a phase combining prompts for narrative episodes and probing questions asking for relevant elaboration and detail. This was followed by a marked transition to questions of evaluation (3), as it demanded the participant to switch to a more reflective-evaluative stance of their experience [46]. The interviews were conducted in the language German. A translated English version of the interview guide is provided in the appendix.

The interviews were carried out by researcher B using online video conferencing software, audio-recorded, and subsequently transcribed. In addition, researcher B wrote a memory protocol immediately after each interview. Eight out of nine guests were included in the study, as one guest could no longer be reached for the interview.

The transcription was carried out using the software aTrain [47]. Relevant passages were subsequently reviewed by researcher A. The duration of the interviews ranged between 36 and 57 min, with an average of 48 min, resulting in a total of 388 min of interview material.

### Sampling

The selection of philosophical practitioners followed a process within the framework of the larger research project. In the first phase, interviews were conducted with philosophical practitioners to explore their experiences with dying, death, and grief. The practitioners were identified via professional associations in Austria, Germany, and Switzerland.

From this sample, we invited practitioners who had experience with these themes or who practiced in palliative care contexts. Seven practitioners agreed to participate (three based in Austria, three in Germany, one in Switzerland).

The selection of guests was organized via invitation or call for participation by the practitioners themselves, which allowed the counselling to take place in the natural setting of each practitioner’s environment. Two practitioners conducted two separate counselling processes.

Informed consent was obtained from both the philosophical practitioners and the guests. In this study, guests—the customary term in this setting, which we also adopt—were invited through the practitioners’ personal and professional networks in community and everyday-life contexts. 

Table 1 provides an overview of relevant characteristics of the philosophical practitioners such as age, previous experience with philosophical counselling and the palliative care context etc.

**Table 1:** Profiles of Philosophical Practitioners

Practitioner	Years of general PP Experience	Estimated number of guests / individual Sessions per year	Experience with Death, Dying, Grief, or Palliative Care	Other Notes
PP_A	24	3 guests / 40 sessions	Works in medical, palliative care and spiritual care contexts; focus on ethics and discussions on assisted dying, and relatives of the dying.	Philosophical practice often mixed with other offers of support; in paid and unpaid arrangements.
PP_C	9	20 guests / 100 sessions	Philosophy seminars on death; as a topic in philosophizing with children; themes of parting, ageing, letting go implicit in individual sessions.	Main professional role as philosophical practitioner
PP_D	16	8 guests / 20 sessions	Focus on death and mourning resulting from biographical experience; offers in hospice and grief counseling contexts; often persons in long-term mourning.	Stronger focus on group sessions, including work with children on grief and death.
PP_E	13	6 guests / nr. of sessions per year not provided	Death and mourning have been a topic since former work as pastoral counselor; trains volunteers in end-of-life and grief care.	Also works as a supervisor and coach; draws on different professional roles in sessions (thus nr. of PC sessions difficult to estimate); overall more group work.
PP_F	21	20 guests / 100 sessions	Former nurse; topical focus in biographical experiences as a nurse; long-standing PP collaboration with senior citizens’ centers.	Focus on series of group-based sessions in hospices and care homes.
PP_G	7	1 / occasional sessions	Experienced social worker (esp. psychiatry), hospice volunteer; deeper engagement with topic and one-on-one sessions of PC based on project.	Philosophical practice with mix form of offers; mainly group and online:

To allow a rough understanding of the background of the guests and the conversational trajectories in the philosophical counselling sessions, Table 2 provides an overview. The first column gives basic information on the guest. The second describes the concrete life situations and subjective experiences which the guests shared as a starting point for the dialogues. The third column lists the (philosophical) themes on which the dialogues centred in relation to these experiences. These short descriptions cannot, of course, convey the depth, dynamics, or complexity of the conversations—that is not the aim of this study. Rather, the purpose is to give a compact impression of the dialogues and the individuals involved.

**Table 2:** Guests of the Philosophical Practice (Pseudonyms), Situations, and Conversation Topics

Name (Pseudonym), palliative care relationship	Shared Core Experiences and Situations	Themes in Sessions
Sabine Mid-60s, experienced physician with background in palliative care	-Increasingly questions own attitudes toward assisted dying -Providing medical care for couples requesting assisted suicide -Reaching retirement age -Having emigrated	Relationality; Freedom; Authority; Shifting values; Resonance; Recognition; Identity; Meaning; Aging; Death
Frida ~ 70, experienced physician in oncology, geriatrics, and palliative care	- Drawing on rich experience from years in palliative care to reflect on how to prepare for dying -Wondering how (early) preparation for dying might look - Social denial of death - Conflicts within the family - Mother’s depression and dementia and death	Forgetting, remembrance, continuity; Preparing for death; A good death; Transgenerational solidarity
Hendrik Early 50s, pedagogue and bereavement counsellor, palliative care patient	- Actively engaging with the question of dying and living well - Living with terminal illness - Others’ exaggerated emotional reactions - Growing self-centeredness - Communicating the experience of approaching death - Speaking truth in the face of mortality	Dying well; Feeling full of life; Unavailability of the self and of life; Assisted suicide; Nothingness; Quantity vs. quality; Letting go; Mourning; Ideals
Maria ~ 60, leadership role in adult education mourning her brother, mother with dementia	- Realizing how much about her brother remained unknown - Wondering whether he needed help - Unable to mourn him with her mother - Facing the loss of her mother soon - Having moved away	Appropriateness; Grief; Care; Closeness and distance; Regret; Guilt; Shame
Magdalena Mid-20s, works in outpatient hospice care, mourning her recently deceased father	- Working in hospice care while unexpectedly losing her father - Balancing organizational duties, mourning, emotional protection, and personal impact - Considering the strained relationship between her partner and father	Remembrance; Grief in everyday life; Consolation; Appropriateness
Ingrid Mid-50s, experienced physician, experiencing symptoms of chronic fatigue	- Questioning standards of established medicine in the light of own experiences with fatigue - Medical vs. personal truth - Alternative medicine - Former backup partner now marrying someone else - Possibly shortened life expectancy due to an unclear diagnosis	Truth; Knowledge; Reality; Right action; Ideals; Orientation; Experiencing finitude; Letting go; Lust for life
Therese ~ 70, retired teacher, recently underwent a life-saving but risky surgery	- Surviving serious illness and surgery - Feeling powerless, exposed, and helpless during treatment - Questioning how life can still be meaningfully filled	Experience of helplessness; Truthfulness; Life review; Meaning; Life after death
Claudia Early 50s, geriatric nurse and psychological counsellor, critical of current practices in institutional care	- Outrage at common practices in geriatric care - Wanting more from life but unsure whether limits are real	Living well: Quantity vs. quality; Meaning; Dignity; Humanity

All names used (e.g., “Sabine”) are pseudonyms. To protect anonymity of the participants, we use coded identifiers (e.g., EZF_1) instead of names. These coded references are also pseudonymous and do not correspond to specific individuals in the tables.

### Translation and language considerations

All interviews, interpretation, and data analysis were conducted in German. For publication, translations from German into English were produced by the authors with AI-assisted support and verified by all authors. We acknowledge that translation is interpretive and may have shaped nuances in the analysis. Examples of challenging terminological choices are as follows. A particularly nuanced German philosophical term was “Geist” (or “geistig”). Depending on context, we rendered it as “mental” when cognitive or psychological connotations were foregrounded, and as “spirit” when spiritual or existential dimensions were at issue. To signal the source term, we retain the German [Geist] in square brackets in the Results where relevant. Furthermore, participants described the effect of the philosophical conversations as “Nachhall”. We chose “lingering resonance” to emphasize an enduring, affective‑intellectual effect rather than (for example) a mere immediate repetition as implied by “echo”. Allusions to Rosa’s concept of resonance [48] are deliberate; while we do not claim a full theoretical alignment, this usage seems consistent with participants’ accounts, which imply an effect on the person and a form of “response”.

### Data analysis

Data analysis followed approaches of qualitative content analysis which combine the use of deductive categories built from the research interest (theory & interview-guide) with inductive category building based on the data [49–51].

Our epistemological stance is interpretivist with a moderate constructivist orientation. We understand themes and categories as analytic constructions developed through interpretive analysis of participants’ accounts. By “moderate constructivist” we mean that participants’ accounts are treated as reports of real experiences, while recognising that our knowledge of them is mediated by language, interaction, and analytic choices. Our multidisciplinary research team combined experience in palliative care and philosophical counselling—in both research and practice—with qualitative methods. We held a dedicated session to discuss researcher roles and, as analysis progressed, routinely revisited them, examined possible sample biases, and challenged our assumptions in ongoing team discussions.

While nine counselling cases were recorded, the qualitative coding and content analysis were strictly limited to the data from the eight cases with completed post-counselling interviews. The process of analysis systematically involved different perspectives to validate the outcome. First, researcher A read researcher B’s memory protocols of the interviews to familiarise with the material and then analysed the verbatim interview transcripts. This involved an initial familiarisation reading of the transcripts followed by a systematic coding process. The interview transcripts constituted the primary dataset and were the sole basis for coding and analysis. Additionally, the practitioners’ reflective protocols were consulted sporadically to contextualize guests’ statements – for example, to check for possible misunderstandings or to understand markedly different perspectives in the experiences of guest and practitioner. These reflective protocols were not coded systematically and did not form part of the primary analytic dataset; they served as ancillary contextual notes.

Second, a joint analysis was conducted with researcher B, who had carried out the interviews, to refine and validate the categories and codes. In a third step, the results were discussed in a workshop with the philosophical practitioners. The findings, representing the guests’ perspectives, were thus analyzed by two researchers and additionally enriched and validated through the perceptions of the practitioners involved.

In sum, the study’s core dataset is multi-perspectival and has, in the process of analysis, been subjected to layered reflection and validation. Given the study’s exploratory aim and the information power of our information-rich sample, we considered the corpus adequate for our questions—without claiming theoretical saturation. The themes we generated from the data are based on material across individual interviews and respective codes were broadly distributed across the dataset. Practical adequacy indicators included a broad distribution of codes across interviews and documentation in a quotation compendium (Supplementary file 2).

In the following section, we present the results of our analysis: insights into the experiences and evaluations of philosophical counselling sessions as expressed by the guests themselves. Quotes were lightly edited for readability (fillers removed; ellipses indicate omissions; clarifying insertions in brackets).

## Results

Our analysis yielded a set of thematic categories, which are presented in Table 3.

**Table 3:** Code Tree: Main Categories and Sub-Themes

	Category	Sub-Themes
1	What is unique about Philosophical Counselling?	a. Stepping out b. A space for free exploration c. Personal involvement and general meta-level d. Questioning and philosophical textse. e. Differences from psychotherapy
2	How is the philosophical practitioner experienced?	a. Benevolent and intellectually stimulating b. Able to navigate the universe of philosophy and communicate as a human being on equal footing c. Listens deeply and contributes thoughtfully
3	What are the effects of Philosophical Counselling?	Effects ‘afterwards’ a. Lingering Resonance b. Seeing differently c. Other practical impacts Effects ‘within’ d. Stepping out e. Inspiration f. Consoling connectedness g. Philosophical curiosity as a stance toward life
4	What are the limits and the strengths of Philosophical Counselling?	a. Foundational attitude and acute psychological distress b. Manipulative philosophical counselling c. Philosophical counselling in societal contexts

### What is unique about philosophical counselling

This category summarizes statements in which guests describe what happens in a philosophical counselling session from their point of view and what they perceive as unique about it.

### a. Stepping out

Guests describe their experience of philosophical dialogue in metaphors, often emphasizing a shift or stepping out. One guest, for example, uses the metaphor of changing tracks:

“[It is] like a second track. A second track alongside it with philosophical keywords that provide inspiration. And then I could – yes, I think that’s a good image – then I could perhaps walk on the other track with [the philosophical practitioner] for a while, but also return to my own track, to my personal involvement. So, very open, as it happened, without a goal” (EZF_6_00:15:35–00:16:03).

Other metaphors also point to this stepping out and entering a specific thinking space. Guests speak of “getting out of a rut” (EZF_6 00:39:50), of taking a kind of a break from the “routine of performative concern,” (EZF_3_00:06:41) of an “island function” (EZF_3_00:07:36), of “looking up at the sky” (EZF_4_00:32:56), or even of shifting of the mind or spirit into “another orbit” (EZF_6_00:34:03).

The metaphor of stepping out does not yet positively define the distinctive nature of the philosophical space. However, the participants repeatedly emphasize the specific quality of this stepping out — and thereby the difference and distance from everyday life and from other conversational formats and modes of thinking — so that our analysis acknowledged this in a dedicated category. In Sect.  3, which examines the effects of philosophical counselling, the phenomenon of stepping out is explored in more detail.

### b. A space for free exploration

While the metaphor of stepping out does not, by itself, define the distinctive nature of the philosophical space, guests described that space as a place for free exploration The following quotes are representative of many similar statements (Further exemplar quotes for this and the following subthemes are provided in Supplementary file 2):

“And that’s what philosophy is really about. It’s not about right or wrong, it’s about exchanging ideas” (EZF_3_00:05:52–00:05:57) It is about “allowing space to let the mind [Geist] and the thinking wander and seeing what else there is to explore” (EZF_4_00:23:54–00:25:07).

Participants highlighted that philosophical counselling offers a space without the expectation of problem-solving or help, and—many noted—without pressure to judge or evaluate. It is a space for open-ended exploration, even when confronting deeply personal existential questions.

### c. Personal involvement and general ‘meta-level’

Guests also reflect on how this kind of free exploration “works”, how they experience the movement of thought in the dialogue. On the one hand, guests emphasize the importance of personal relevance; otherwise, the conversation remains too intellectual or distant:

“When you talk about death without really being affected, it’s much easier. It stays abstract, it doesn’t touch you. But when you *are* affected […] it becomes a whole different thing. It’s personal” (EZF_2_00:13:57–00:14:23).

This openness—to personal, even painful experiences, and to deep reflection—is seen as key to making the dialogue meaningful. On the other hand, what is unique about philosophical counselling is that these felt experiences are taken to another level—a “meta-level” (EZF_2_00:05:52; EZF_7_00:13:22), as many guests called it:

“What stood out to me as the main difference in a philosophical conversation is that one tends to talk about the topic more theoretically, working less directly on the topic itself, but rather […] philosophizing a lot about hypotheses. That one essentially questions one’s own thought constructs as such […] asking, why do I even think in this particular hypothesis about the topic in this way?” (EZF_2_00:04:57–00:05:36).

The guests describe a pendulum-like movement—or an interweaving—between very concrete experience and reflecting on it at a meta-level that involves a certain distance. This linking of the concrete and the general, and the dual willingness both to disclose something of oneself and to adopt theoretical distance in order to speak about it in general terms, characterizes philosophical conversation.

### d. Questioning and philosophical texts

Guests also identified “methodological” elements that, in their view, are equally constitutive of philosophical inquiry. They named the questioning of assumptions, the inclusion of texts, the search for and reflecting on concepts, and the integration of theory and different perspectives as distinctive features of philosophical counselling. Texts—whether brought into the sessions as quotes or read between sessions—often took on special significance:

“And the philosophical practitioner provided me with a wonderful text. Based on a conversation, they had this idea. […] And the text inspired me again—it really stayed with me; it had a lasting impact” (EZF_3_00:26:29 − 00:26:59).

Most strongly emphasized, however, was the act of questioning itself. Guests experienced this kind of questioning as profound, deepening, and often critical:

“First, she just let me talk or asked if there was anything on my mind, and I usually had a topic with me, so to speak. I would just talk and talk and talk, and then at some point, she would ask follow-up questions—really profound follow-up questions […], critical questions. And for me, at least, when I get a critical question, I sometimes think: Oh! Okay… And then you start thinking in a different way, so to speak. And I found it really impressive how she did that—very subtle, but still effective” (EZF_4_00:05:54–00:06:40).

### e. Differences from psychotherapy

A key finding of this study is that guests consistently perceived “a very clear difference between philosophical counselling and psychotherapy” (EZF_5_00:17:54). As one guest put it:

“It’s not therapy. It’s not meant to be. It’s really more of a conversation sparked by ideas. When I deal with death and grief, that’s not a medical issue. It’s something else” (EZF_6_00:32:34–00:33:41).

In our sample, participants identified two main differences from psychotherapy: philosophical counselling is not a help‑oriented service—and our guests did not expect it to be “therapeutic”—and there is no requirement to solve problems, that is, no pressure to act. Taken together, these features were seen to afford greater freedom of thought in philosophical counselling.

### How is the philosophical practitioner perceived – what do they do?

The descriptions of what characterizes philosophical conversation are closely linked to how the philosophical practitioner was experienced by the guests. Many guests described two poles that were not strict opposites, but qualities held in a productive tension.

### a. Benevolent and intellectually stimulating

The philosophical practitioners in our sample were consistently described as both empathetic and intellectually engaged—human and deeply rooted in philosophy. One guest called their practitioner “benevolent” and “a bit intellectual — but in a pleasant way” (EZF_6_00:36:17 − 00:36:30), another described them as “a counterpart who is kind and curious about what you think” (EZF_7_00:01:09); another guest said that what the practitioner does is “intellectually demanding” (EZF_8_00:01:54), that [they] are “highly competent” (EZF_8_(00:07:11), and at the same time “so gentle” (00:07:08) as a person.

While intellectual acuity and benevolence are not mutually exclusive, participants suggested that they experienced philosophical practitioners as highly intellectually rigorous, *yet* deeply empathic and attentive listeners. This balance was notable for several participants.

### b. Able to navigate the universe of philosophy while communicating as a human being on equal footing

Closely related to the idea of benevolent intellectuality—which seems to describe an overall attitude or presence—is this combination of deep philosophical knowledge and accessible communication. According to the experiences of our guests, it is essential “that that the practitioner is deeply read in philosophy” (EZF_8_00:17:28). However, it is not about lecturing or promoting one particular philosophical school. Rather, this breadth of knowledge becomes part of a personal philosophical culture, which is what makes it effective.

This often created a sense of mutual exchange: “What fascinated me most was how it turned into a real give and take. […] One of my thoughts, one of hers, and it grew from there—into something bigger, deeper. I found that very beautiful” (EZF_5_00:07:58 − 00:09:56).

Guests did not see the practitioner as someone performing a professional service, but as a human being—as someone who is also searching: “She didn’t switch into some kind of professional role. The way she philosophizes is very connected to who she is as a person” (EZF_8_00:19:28–00:19:46).

Participants seem to assume that, in general, there is a tension between philosophical erudition and relating at eye level, but they did not experience it with practitioners in our sample; practitioners were perceived to balance it effectively. Participants attributed this to encountering the practitioner as a person rather than solely as a role-bearer, with philosophy enacted as a personal stance.

### c. Listens deeply and contributes thoughtfully

Guests emphasized aspects of how their practitioner listened. Practitioners were described as attentive listeners who, while staying close to what was said, would also paraphrase or highlight key terms and expressions, helping guests become aware of deeper meanings. This often made space for philosophical reflection, even before explicitly introducing questions, texts, or theories.

The following quote combines many aspects that were often mentioned separately in other interviews: listening with sensitivity, and at the same time questioning, introducing different perspectives, including philosophical texts—all done in a style that is both empathetic and critical:

“Yes, the [philosophical practitioner] asked questions and also challenged some of the things I said—whether in terms of content or whether there might be another perspective to consider. [They] sometimes brought in quotes or philosophical excerpts, which I found very inspiring as they encouraged me to reflect and engage with new ideas. This often introduced a different aspect to the conversation. I also found [them] very empathetic, not controlling, but rather skillful in a positive way. Not manipulative—absolutely not—but striking a good balance between responding to me and, through her questions, introducing new perspectives or challenging certain points, like, ‘Couldn’t this also be seen differently?’ I thought this mix was excellent” (EZF_5_00:13:53 − 00:15:18).

Participants also perceived a potential tension between critical interventions and a listening stance. In our sample, participants noted as remarkable when practitioners posed critical questions and perspectives while maintaining a listening, receptive stance.

### Effects of philosophical counselling

The guests’ reported effects of philosophical counselling are grouped into two sub-categories, each with specific sub-themes. Guests describe (a) effects that manifest after or between the sessions (“after-effects”), and (b) effects that the sessions generate as a value in themselves. While this distinction is not always clear-cut, it is important to highlight the intrinsic value that participants attribute to the conversations.

### a. Effects ‘afterwards’

#### Lingering resonance 

One participant summed up how the sessions left a lingering impression in everyday life, even if this could not always be precisely articulated:

“[They are] interesting conversations in the moment itself, as they happen, and then a kind of lingering resonance that somehow sticks with me. Or that resurfaces on other occasions […] a bit like sustainability: I’m not constantly mulling over what was discussed, but things do come up again in the back of my mind” (EZF_6_00:00:57–00:01:25).

Thus, the outcomes of philosophical conversations are not immediate actions but rather seeds of thought planted during the sessions “that might unfold later” (EZF_2_00:21:30–00:21:45).

Lingering resonance means that, rather than reappearing only as a straightforward memory, certain thoughts continue to work on participants in daily life, sometimes eliciting a response or catalysing further development.

#### Seeing differently

This lingering effect becomes more concrete when participants describe how the conversations allowed them to “see things differently”. For example, one guest noted that the conversations were “a challenge for me, but I grew a lot from them, because I came to realize […] that I am very capable of changing my perspective” (EZF_1_00:01:42 − 00:01:58).

This new way of seeing is linked to personal growth, particularly in terms of clarity and inner freedom. It results from both a broadening of perspectives and a disruption or questioning of one’s own often implicit assumptions.

#### Other practical impacts

Beyond the valuable yet somewhat vague sense of “lingering resonance” and the more defined change in perspective, participants also reported tangible, practical impacts: increased engagement with philosophy; motivation to read more; support in decision-making; inspiration from specific texts that influenced their work; and a sense of possibility for personal growth.

### b. Effects ‘within’

When speaking about the effects of the philosophical conversations “in themselves”, participants often used broad, emotionally charged terms to indicate that the sessions were good or relevant. For instance, one interviewee said they were “touched on the inside” (EZF_1_00:02:39), another said, “[…] this is currently the best kind of therapeutic support I can open up to” (EZF_3_00:02:27). Three core characteristics became apparent:

#### Stepping out

The first key feature of why philosophical conversations are experienced as intrinsically valuable is what has already been described earlier as “stepping out”. The description of “stepping out” has been provided above, and we will therefore focus here on the effects implied by it. When we summarize—drawing together what is described across the results presented here—what, in the perception of our participants, they are stepping out of, the distinctive contours and effects of the philosophical space begin to take shape. Philosophizing, in summary, allows for a certain kind of *distancing*, a “stepping out” of:

(too) deep personal involvement and affection,
what is perceived as the usual or familiar way of speaking about existential experiences in psychological terms or narrating them,
everyday judgment – participants repeatedly highlighted the liberating absence of value-judgements in philosophical practice,
the pressure to act or to solve a problem as well as from the practical routines and expectations of everyday life,
the narrowness of habitual thought patterns.

Thus, “stepping-out” refers to, as reconstructed from participants’ accounts, a temporary shift into a distinct philosophical space—described as “another track,” an “island,” or “another orbit”—in which distance is taken from usual involvements (especially action orientation and strong affective involvement) and from familiar ways of speaking (focused on narrating and problem-solving), opening a less habitual, more reflective and exploratory way of addressing things. It is defined primarily ex negativo (a stepping out of), yet experienced as intrinsically valuable in its own right.

#### Inspiration

This stepping out is seen as inherently valuable. Participants used various expressions to describe the experience of inspiration that arose from it (emphasis added by the authors):

- “I experienced it as very *joyful*—so *beautifully enriching*” (EZF_7_00:01:59).
- “That was what made it so *fascinating*. After supervision, I often feel motivated, but also exhausted—it’s work. You work to solve a problem. And in Philosophical Practice, it was never the case that I left feeling exhausted, but rather always *more awake*—*awake in spirit [Geist]*” (EZF_4_00:12:50–00:13:13).
- “I’ve become much *more conscious* about themes like life, dying, and mortality” (EZF_1_00:02:28-).
- Another participant spoke of “*awareness* of life, *wisdom* […] letting in *fresh air*” (EZF_8_00:08:02).

The idea of the *mind* or *spirit* [“Geist”] was a recurring motif. Participants spoke of their inner spirit [“Geist”] being allowed to wander freely. The term “inspiration” does not appear in vivo in the data but seems an appropriate way to summarize what the interview participants conveyed in various ways.

#### Consoling connectedness

Many participants spoke of a sense of reconciliation or comfort that grew out of the conversations. This sense of comfort was often described in terms of *connectedness*. The idea that *others*–thinkers across time–have asked similar questions and faced similar fears, evokes a deeper, more universal form of connection:

“[…] I ask myself: what does the world say about this? Who has something to say? That’s a relief. It’s like a door opening to a land where you realize: oh, someone has already been here. And they saw it this way, and others saw it that way. And that’s comforting – not in a therapeutic way, but still very soothing” (EZF_8_00:14:16–00:14:45).

There seem to be various levels or forms of consoling connectedness. Some of them are not necessarily specific to philosophical conversations, such as the feeling of being seen or not being alone with one’s thoughts and emotions. While these forms of connection can occur in other types of conversations, participants also described a possibly uniquely philosophical form of connectedness: a kind of solidarity with humanity, through shared reflection on the human condition. This suggests a kind of philosophical sense of *not being alone in one’s humanity*.

#### Philosophical curiosity as a stance toward life

Our interview partners suggested that philosophical curiosity—the practice of asking questions and engaging in reflection without instrumental goals—must be somewhat present in a person already to be receptive to philosophical counselling. At the same time, this attitude can also evolve and become a guiding principle in one’s professional and personal life, particularly in care professions or when facing one’s own mortality. This attitude becomes a possible way of facing death. As a final note, we give the last word to one serious ill guest, who embodies this philosophical stance even as death draws near:

“Someone asked me if I had reached some kind of wisdom in aging or dying. And I thought—wow, that’s a big one. And then I said: Yes—stay curious!” (EZF_3_00:37:13–00:37:25).

### On the limits, ambivalences and strengths of philosophical counselling

#### a. Foundational attitude and acute psychological distress

When asked where the guests would draw the line for philosophical counselling—that is, in which situations it might be unsuitable or even harmful—a consistent answer was that philosophical dialogue becomes impossible when someone is under acute psychological distress. A certain degree of inner stability or at least some basic grounding is required. Most of these statements were expressions of personal opinion and appraisal. Yet, the following example refers to direct experiences from the counselling sessions and reflects on the potential destabilizing effects of philosophical reflection. However, this destabilization is described as ambivalent:

“In that moment, it briefly felt somewhat threatening to me. And that was perhaps where I’d say there’s a bit of a boundary for me: that my mental constructs aren’t challenged too much, because I simply need some of them. But even so, I’m glad when I’m offered opportunities, so to speak, to think differently” (EZF_2_00:18:47 − 00:19:11).

In our sample, no boundary was crossed. The philosophical practitioners either refrained from overly critical probing (returning to a listening mode) or—according to the guests—introduced critical questions with humour or in other skilful ways. In this manner, the questioning could still be experienced as productive.

#### b. ‘Manipulative’ philosophical practice

Our interviewees repeatedly emphasized that the philosophical practice they experienced in this study was consistently open, free, and grounded in dialogue. But they also expressed concerns about what could go wrong if this quality is lost. One interviewee warned:

“It would also be a problem if a very dominant person only wanted to impart their knowledge […] That person belongs in a lecture hall” (EZF_8_00:25:11 − 00:25:27).

Another concern is that philosophical counselling might degenerate into a kind of standardized program, serving the optimization of dying. Philosophical counselling must retain its freedom and critical distance. In summary, philosophical counselling should retain openness and critical freedom; avoid didacticism and asymmetry.

#### c. Philosophical counselling in societal contexts

For completeness, our interviewees also reflected on philosophical counselling in societal contexts. Their reflections included the sceptical question of whether philosophizing might be only for the educated middle class, while at the same time emphasizing its relevance for the healthcare system and society:

“That is something important for the care sector, and it’s almost even more important in society to say that we really need many more spaces to talk about things without the aim of being right or proving someone right […] but rather to have a space to exchange: what do I understand, what do you understand, and what do we do with that now? I find that very moving as well, because I’ve realized that we simply need much more of this” (EZF_1_00:27:10 − 00:28:10).

The hypothesis voiced by guests, based on their experience, is that both the healthcare system and society need spaces for free exploration and for stepping out in the sense described above—spaces in which people can meet one another in thinking, as human beings, without further purposes (such as help or decision-making).

### An illustrative composite vignette

To give a concrete sense of what a 60–90-minute session of philosophical counselling can feel like—and to concisely synthesise the elements presented in the Results—we provide an illustrative, ideal-typical vignette. The following vignette is a composite built solely from guest interview excerpts and does not draw on session transcripts, in line with the study’s dataset and analytic focus. Although it consists of verbatim guest quotes, it is a constructed collage drawn from different interviews. We offer it to help readers grasp the ideal-typical features in a compact way. Brief narrative cues are added to stitch the excerpts together without altering the quotations. For readability, source identifiers are omitted here; all quotations are verbatim and traceable in the Results and the quotation compendium (Supplementary File 2).

#### Illustrative composite vignette

The session often begins with easing into speech and attentive listening: “I start by just talking—what’s on my mind—and she listens, stays close to my words. Then a gentle but critical follow-up lands: a question that makes me pause and think differently.” As the exchange unfolds, guests notice a shift toward exploring the phenomenon on a more general level: “What stood out to me as the main difference in a philosophical conversation is that one tends to talk about the topic more theoretically, working less directly on the topic itself, but rather […] philosophizing a lot about hypotheses.” Within this frame, normative pressures temporarily recede: “what one should, must, or could do … doesn’t matter.” Texts may be introduced and often have a particularly inspiring effect: “And the philosophical practitioner provided me with a wonderful text. Based on a conversation, they had this idea. […] And the text inspired me again—it really stayed with me; it had a lasting impact.” Across the process, the philosophical space itself is experienced as a kind of stepping‑out: “getting out of a rut … ‘island function’ … ‘looking up at the sky’ … ‘another orbit’”. As a practice, philosophical counselling is experienced as permissive and non‑directive (especially compared with psychotherapy or supervision): “very free … no judgment … less directive.” As persons, practitioners were described as empathetic and intellectually engaged—human and deeply rooted in philosophy: “A counterpart who is kind and curious about what you think.” Conversations often have lingering resonance and inspire further reflection: “I leave not exhausted but more awake—inspired, with a bit more room inside to see differently.”

## Discussion

### a. Limitations and strengths of the study

This study’s main limitation is its small sample size; nevertheless, it is exploratory and, to our knowledge, the first to foreground the perspective of guests of philosophical counselling in palliative care, adopting a client-based evaluative stance rather than the autoethnographic or practitioner-led perspectives common in prior work.

Moreover, each of the eight interviewees shared their perspective based on five sessions with experienced philosophical practitioners, many with familiarity in palliative and existential issues. The guest sample additionally represents information-rich expert sampling: guests were also experienced in dialogue, with many backgrounds in psychotherapy, supervision, or communication-related professions.

While this likely enhanced the quality of their assessments, it limited diversity; as some guests themselves asked whether philosophising might be only for the educated middle class, future research should include more diverse participants and contexts. This pertains not only to social and educational status; our study also cannot speak to how philosophical practice might look when guests face additional constraints due to illness or, for example, neurological or cognitive challenges—indeed, the guests in our study tended to be rather articulate.

It is also important to note that practitioners selected the guests themselves. An advantage is that the guests came from the practitioners’ natural lifeworld (i.e., such encounters could have arisen without the research project, and in some cases did), but this introduces selection bias. Guest feedback was consistently positive, highlighting the quality of dialogue and practitioner–guest relationships, while challenging or failed dynamics were not captured; guests appeared favourably disposed toward the project and the practitioners, and practitioners may have chosen conversation partners with whom “little could go wrong,” making the conversations perhaps “too successful,” and they may have been especially keen to create a safe environment.

We consulted practitioners’ brief reflection notes to contextualise the findings (these were not analysed as a separate dataset), which yielded two summary impressions: first, the conversations do not appear atypical, suggesting the project did not produce unduly artificial situations; second, practitioners sometimes felt the need to justify sequences they viewed as less successful or “not philosophical enough,” often because they had given guests room simply to tell and were themselves listening—for instance, when grief was strong, it did not seem appropriate to push a philosophical meta-level or critical questioning. This accords with guests’ needs and perceptions but also reflects a bias insofar as most practitioners in our study worked within a broadly hermeneutic frame, emphasising stance, relational quality, and a safe setting.

Finally, while tentative, our findings converge with emerging research on philosophical counselling in palliative contexts: the foundational significance of a ‘sense of wonder’ [39], which in our study was often described as a stance of curiosity and openness; the role of hermeneutics as a conceptual and ethical framework [24]; the ‘relief’ that comes from stepping out of the role of ‘being helped’ [43]; and the ‘courage’ to face problems not to resolve them too quickly, but ‘to stay’ with them and cultivate a creative environment that makes ‘space for the other and for the new’ [40].

### b. Value and meaning of philosophical counselling

#### Participants’ perceived value of philosophical curiosity

Our findings indicate that, for some participants, philosophical counselling spoke to a genuine interest in reflective depth and existential inquiry that is distinct from therapeutic or religious frameworks. Participants valued the freedom to explore open-ended questions without the pressure of achieving specific goals or symptom relief (“nothing has to be solved”). Participants sought to connect personal experiences with broader philosophical and human concerns.

We developed the theme of philosophical curiosity as a fundamental attitude toward death and dying, offering grounding in the face of existential vulnerability. This approach provides freedom, clarity, and consolation through reflective engagement with life’s profound questions. Participants reported a sense of self-understanding and connection to the mystery of existence.

Since Aristotle, wonder has been regarded as a basic emotion and the beginning of philosophical consciousness and thinking. The relationship between philosophical wonder and religiosity and spirituality has also been developed in practice-oriented ways in the philosophical literature [52, 53]. In the Spiritual Care discourse, while the “influence of reverent wonder on well-being” is aptly discussed [54], explicit philosophical references are not elaborated.

In this connection, our findings broadly align with Hansen’s comprehensive work on the significance of wonder in philosophical practice [39]. By wonder we mean an experience and stance of openness and receptivity to the matters of life (and to what others say), one that resists subsuming encounters under prior knowledge and allows itself to be touched by not‑knowing (cf. Lindseth [24]; Hansen [39]).

In his phenomenology of wonder, Pöltner [55] stresses that wonder does not add information or new facts; rather, one is seized by the sheer “there is”—that anything appears at all, and in the way it appears, including the very possibility of questioning. Participants in our study seemed, first, to experience the philosophical space in precisely this quality and, second, to regard it as a viable existential stance.

Whereas in Hansen [39] wonder often bears a distinctly spiritual tonality, the largely convergent “philosophical curiosity” and “openness” reported by our participants also carried an additional, arguably civic or political, note: openness to other perspectives, a readiness to question one’s own assumptions, and even to be refuted. Our interviews thus suggest not only existential but also sociopolitical relevance. These facets of wonder warrant further exploration; the basic direction is sketched here.

As spiritual care in healthcare is often tied to religious or psychotherapeutic traditions, the need for philosophical practice remains underrecognized. This study calls for expanding existential support to include non-directive, dialogical formats rooted in philosophical openness, which participants found both valuable and consoling.

#### Consolation, connection, and reconciliation – in a distinctly philosophical mode

Connectedness is a key dimension of spiritual experience and development for people at the end of life, in bereavement, and in caregiving [56, 57]. Participants in our study described relief through connection, first in this psychological sense: conversations help one feel seen, articulate fears, tell one’s story, and be met by an empathic listener. We might call this a form of psychological connectedness, corresponding to common non-specific factors in helping conversations. The practitioners in our sample evidently enacted an empathic stance in dialogue. This is desirable in this context, but it is not specific to philosophical counselling.

Beyond these non-specific relational factors, guests in philosophical counselling also found consolation in the philosophical tradition itself—feeling part of a lineage of thinkers who have grappled with mortality and meaning. This experience may foster a sense of solidarity, as one participant put it: reading or hearing what “others have thought” feels like “a door opening into a country where I’m not alone.” This form of connectedness seems to differ from the psychological one. In the EAPC definition of Spiritual Care, connectedness plays a central role: spirituality is defined as a dynamic dimension of human life—independent of religion—that relates to how individuals seek meaning, purpose, and transcendence, and how they connect to the moment, self, others, nature, or the sacred [58]. Our guests appeared to report less a connection to self, others, nature, or the sacred than to “humanity” and the experience of a shared existential condition. This tentatively raises the question of whether philosophical conversation may enable a distinctive form of connectedness that otherwise tends to be overlooked. In our study, we find indications in this direction. Philosophical counselling, to put this cautiously and as a hypothesis, thus may offer a reconciliatory experience of human solidarity—intellectual, emotional, and existential—providing a quiet yet potentially profound response to the loneliness of dying or to existential isolation [59].

### c. Qualitative dimensions in philosophical counselling within palliative care

While further research and implementation studies will be needed to define quality standards in philosophical practice more generally, the present study provides coherent—albeit preliminary—insights into the qualities and conditions that may characterise responsible practice in palliative care and hospice work. A recent key contribution to the question of quality in philosophical practice has been provided by Romizi [60]; we refer to this, as well as to the three foundational styles of philosophical practice introduced at the outset, to relate our exploratory findings and this field to that discussion.

#### Dialogical style and ethos of philosophical counselling in palliative care

As outlined in the introduction, the field of philosophical practice can be broadly distinguished into three styles: the hermeneutic‑dialogical style, the critical‑thinking style, and the contemplative style. In this sample, practitioners predominantly employed a hermeneutic‑dialogical approach, characterised by open‑ended questioning, deep listening, and co‑exploration of meaning. This style avoids fixed methods and emphasises the practitioner’s personal presence, including their own existential experiences. Critical analytical interventions were present but were carefully embedded both within an empathic relationship and, for the most part, within ample space for extended narrative. Overly critical questioning was experienced as potentially destabilising; on the other hand, guests also found these “critical” aspects fruitful and genuinely philosophical. This may suggest that, with vulnerable themes and situations, caution in critical thinking is important; yet if it is absent altogether, the “philosophical” may be lost.

Against this backdrop, Romizi [60] describes, in the form of an axis within a matrix, that good philosophical counselling is marked, among other things, by a balance between a hermeneutic and a critical stance. The conversations as experienced by our guests appear, within this schematic, to tend towards the hermeneutic pole; at times—when existential strain was high—critical aspects were sometimes set aside entirely to make room for telling and listening. At the same time, our study also suggests a slightly different picture that may not fit neatly into a scale or matrix: critical thinking seems, rather, to be *embedded within* a hermeneutic practice and frame.

Turning to the contemplative style, it may be noted that none of the conversations contemplated philosophical texts strictly according to Ran Lahav’s method [32]. However, guests often valued the use of philosophical texts, and texts often generated a “lingering resonance,” that is, a deeper after‑effect at which the contemplative approach, in substance, aims. This suggests that integrating contemplative text work could, where appropriate, enhance sessions by resonating intellectually and emotionally.

There are plausible reasons why the hermeneutic style may have been foregrounded. First, the German‑speaking tradition of philosophical practice—shaped by Achenbach (and Lindseth)—has long promoted open dialogue, up to and including a reluctance to speak of “method [18, 19, 60]” Romizi [60] also observes a distinction between Anglo‑American and continental orientations in philosophical practice: the former tends to be more person‑oriented as a kind of help (cf. the well‑known book-title by Lou Marinoff, “Plato, not Prozac!” [61]), at times even positioning itself as an alternative to psychotherapy; by contrast, the continental orientation tends to be more “matter‑oriented,” as a free exploration of fundamental life themes. Our findings appear to align with the continental orientation. Second, the ethos and ethics of communication in hospice and palliative cultures tend to exhibit a similar basic stance—prioritising empathetic presence, careful pacing, and safety—which philosophical practitioners in our sample appeared to realise. Moreover, as described in the Methods, our sampling included practitioners already familiar with end-of-life themes—and in some cases with palliative settings—which may also have favoured a hermeneutic emphasis. Third, it may simply be an appropriate way to philosophise in the face of death and moving existential questions: philosophical practitioners seemed to enact a communicative stance that closely resembles established good practice in palliative care and hospice work.

#### The philosophical practitioner

In Romizi’s model, [60] considerations of quality in philosophical counselling are developed with reference to methodological elements of the conversational process; the person of the philosophical practitioner is not directly in view. In our study, guests also reported at length—of course, we had explicitly asked about this—how they experienced the practitioner. It appears that the quality of philosophical counselling may also be experienced as arising, at least in part, from qualities of the practitioner that do not lie in methodological moves or procedures, but in the practitioner’s stance and the resulting quality of encounter and atmosphere, as reported by guests in this sample. In the Results section, we tentatively interpreted these as a balance or synthesis of poles that are not logical contradictions, but are often held in productive tension.

The “virtues” of philosophical practitioners, as described by guests as embodying such a balance, include (a) a stance of “benevolent intellectuality” as a core attitude (intellectually rigorous yet kindly), (b) a combination of erudition and, so to speak, communicative modesty—rootedness in philosophical tradition while communicating on an equal footing; this also includes a listening habitus, (c) the skill of deep listening combined with thoughtful contributions, and (d) existential self‑concern, grounding their presence in shared human vulnerability.

Crucially, practitioners were, for the most part, described not as playing a role or delivering a service, but as present, authentic individuals—fellow seekers who brought their own existential questions into the dialogue. By acknowledging uncertainty and engaging in co‑thinking rather than instructing, they seemed to foster a genuine atmosphere for reflection—i.e., a philosophical space. These practitioner‑centred virtues may complement Romizi’s method‑focused model by highlighting how stance and presence contribute to perceived quality.

#### Preserving the philosophical space

Guests emphasized that the value of philosophical practice lies in intellectual freedom within genuine human encounter. Quality assurance should preserve this free space and avoid hidden goals, such as providing help, promoting normative ideals, or optimising outcomes.

Philosophical Counselling may create such a free space for conceptual exploration, reflective dialogue, a personal–universal balance, and goal-free openness. This values exploration for its own sake, which—paradoxically, and as an unintended side‑effect—may lead to clarity or transformation, a felt sense of connectedness, and inspiration.

An important criterion for identifying a conversation as philosophical, according to Romizi,Nr. 60 is “anti‑psychologism”: the exchange attends less to underlying psychological mechanisms or to the interlocutors’ psychological well‑being, and instead connects individual concerns, experiences, and questions with more general concepts, ideas, and questions. In our study, guests particularly highlighted and valued this feature. This may be surprising, given that in a context where suffering and relief, need and help are so prominent, one might have expected philosophical conversation to be understood as another form of helping talk.

On the contrary, its specific value in palliative contexts may lie not in adding yet another help‑oriented format, but in offering an open space in which the “spirit” [Geist] can be tended and open questions explored for their own sake—an intrinsic need that does not require reduction to other aims. Rather than speaking in negative terms of “anti‑psychologism,” one might also adopt a positive label aligned with guests’ wording, such as “free exploration of ideas,” where the implicit contrast is with any instrumental “processing” of personal‑psychological states or problem‑solving as the primary aim.

To preserve openness and freedom, we would further suggest that quality should not rely primarily on certification or standardisation but emerge from good practice—dialogical attentiveness, intellectual integrity, and ethical sensitivity. As in philosophy’s best traditions, quality grows through mentorship, reflective engagement, and social learning within a community of practice, rooted in ongoing *Bildung* and dialogical responsibility [62, 63].

### d. Philosophical counselling in a public health approach to palliative care

Our interview partners suggested there may be a need for more spaces of open, non‑instrumental exploration of fundamental life questions, coupled with genuine interpersonal encounter in our society and in the health system. In a broader perspective, such reflections could be linked to public health approaches to palliative care. The Lancet Commission on the Value of Death [14] notes that how societies engage with death, dying, and mourning is entwined with their philosophical traditions; reintegrating death into life requires addressing questions of meaning, value, and the human condition—the core remit of philosophy. Conversations and stories about everyday death, dying, and grief should become common. Here, Death Cafés and Last Aid Courses are cited as conversational spaces; interestingly, there is no reference to explicitly philosophical formats. To this discussion one might add that philosophical counselling offers a dedicated space to explore existential questions and to situate personal experience within wider social and intellectual contexts.

For future development and research, the question may be raised whether—and in what ways—by exploring and critically examining one’s relationship to ultimate questions, philosophical counselling could contribute to facets of death literacy, particularly if death literacy is understood not merely instrumentally but as practical wisdom [12, 64, 65]. Looking ahead, we encourage creative experimentation with philosophical conversations on life and death, not prescriptively guided but inspired and informed by our study’s findings and the associated quality considerations.

### e. Future research directions

Building on the exploratory nature of this study, further research into Philosophical Counselling in palliative and existential contexts could pursue at least three strategies:

1. A broad-based, mixed-methods survey of guests, combining quantitative and qualitative elements, focused on philosophically grounded qualities and outcomes.
2. Practice-based implementation studies in hospices, care teams, hospitals, and community settings.
3. The conceptual development and empirical evaluation of quality criteria that preserve freedom, non‑instrumentality, non‑standardisation, non‑trivialisation, and the possibility of radical critical questioning.

## Conclusions

Philosophical counselling seems to offer a space for reflective depth and existential inquiry in palliative care, fostering curiosity, wonder, and connectedness. Its value lies in intellectual freedom and an open, goal-free space. Practice implications include preserving this space, resisting help-driven agendas and didactic or overly abstract modes, and calibrating critical inquiry—embedded within a hermeneutic frame—inside empathetic dialogue. Philosophical counselling may contribute to facets of death literacy, understood not merely instrumentally but as practical wisdom. While our exploratory sample is context-specific, our findings suggest that end-of-life themes are already being addressed within philosophical counselling and offer cautious guidance for introducing and integrating such practice alongside existing psychosocial and spiritual supports in palliative care contexts.

## Supplementary Information

Supplementary Material 1.

Supplementary Material 2.

Supplementary Material 3.

Supplementary Material 4.

## Data Availability

Due to the sensitive nature of the topics discussed (death, dying, and bereavement) and the potential for deductive disclosure within the small professional community of philosophical practitioners, the data for this study are not publicly available to protect participant privacy and professional confidentiality. Data may be made available from the corresponding author upon reasonable request, subject to additional anonymization and ethical considerations.
