# Supplementary material for: ‘Stay Curious’: guest experiences of philosophical counselling in palliative care – a qualitative study
Source: BMC Palliat Care. 2026 Jun 29;25:271. doi: 10.1186/s12904-026-02178-x (PMC13576289; doi:10.1186/s12904-026-02178-x)
Supplement: Supplementary file 2 [file 12904_2026_2178_MOESM1_ESM.docx]

**Addional File 1 – Interview Guide (English version)**

| **Phase** | **Procedure/Prompt/Questions** |
| --- | --- |
| **Introduction** | A. **Introduction**    A.1. **Researcher introduces themselves**    A.2. **Thank for participation**    A.3. **Explain context of the study**    A.4. **Ask for recording (start recording, if yes)** |
| Experience | B. The experienced Philosophical Counselling [PC]    B.1. Starting broadly, how did you experience in the PC sessions with [practitioner name]? What can you tell us about it?    B.2. What was your relationship to the practitioner? What led you to them (already known, regular/new guest, by advertisement, private relationship, work colleague, …) C. Motivation to participate and relation to dying, death, and grief    C.1. The counselling conversations took place within the framework of this study on the role of philosophical practice in the context of palliative care and hospice work. What is it that motivated you to participate in this study?    C.2. Philosophical practice is overall not very well known. What expectations did you have? D. Actual procedures    D.1. What then actually happened in the PC sessions? If you would have to explain to someone in three sentences, what one does in a PC session, how would you describe it?         D.1.1. What is it that the practitioner does (as concrete as possible)?         D.1.2. What is it that you as “guest” does (as concrete as possible)? |
| Evaluation | E. Evaluation of experience    E.1. If you now look at your experience, what would you say is similar or different to what you have known already?    E.2. What would you say is specific about PC? F. Impact and remember moments    F.1. When you think back to the PC conversations, was there a moment you remember particularly well or that means something to you?    F.2. If yes:         F.2.1. please describe this moment and scene of the PC sessions briefly.         F.2.2. What is it that makes this moment so memorable/particular?    F.3. Overall: What do you take away from the PC sessions?         F.3.1. Probing question, if applicable: How would you describe this impact or effect? OR: What is different now (as compared to before)?    F.4. Considering the context of our study we are also interested in: How did the topics of death, dying, and mourning touch you in the conversations?         F.4.1. Independently from the concrete topics: What effects did the philosophical engagement with finitude had for you personally, as a human being/concretely/in your everyday life? |
| Closing with factual biographical | G. Closing    G.1. Is there a message you would like us to consider in the research project?    G.2. Would you like to give any feedback to the practitioner? H. Biographical information    H.1. Pseudonym    H.2. Age    H.3. Gender    H.4. Geographical area of residence    H.5. Attended PC with    H.6. (Previous) relationship with practitioner    H.7. Professional background    H.8. Educational background    H.9. Previous experiences with philosophy |
