# Supplementary material for: ‘Stay Curious’: guest experiences of philosophical counselling in palliative care – a qualitative study
Source: BMC Palliat Care. 2026 Jun 29;25:271. doi: 10.1186/s12904-026-02178-x (PMC13576289; doi:10.1186/s12904-026-02178-x)
Supplement: Supplementary file 3 [file 12904_2026_2178_MOESM2_ESM.docx]

**Additional file 4: Extended exemplary quotes by theme**

| **Category** | **Sub-Theme** | **Quote** |
| --- | --- | --- |
| **1** | Stepping out | Guests speak of “getting out of a rut” (EZF_6 00:39:50), of taking a kind of a break from the “routine of performative concern," (EZF_3_00:06:41) of an "island function" (EZF_3_ 00:07:36), of “looking up at the sky” (EZF_4_00:32:56), or even of shifting of the mind or spirit into “another orbit” (EZF_6_00:34:03). |
| **1** | A space for free exploration | "And such things simply don’t matter at all with [the philosophical practitioner]—what one should, must, or could do, or anything like that. Not at all. So [it’s also] interesting that in everyday conversations, you’re very quickly confronted with advice or recommendations for action [...] And I think it often comes from an impulse to help [...] Maybe that’s precisely why Philosophical Practice is such a space where you’re allowed to simply explore." (EZF_6_00:19:27 – 00:20:33)  This matter was also expressed as follows: philosophy “is not about right or wrong, it’s about exchanging ideas” (EZF_3_00:05:52 – 00:05:57) |
| **1** | Personal involvement and general meta-level | This also implies a readiness to express what one fears: "That you speak about the things you’re afraid of—that’s part of showing yourself" (EZF_3_00:19:59).  This principle of personal resonance applies not just to experiences, but also to the ideas discussed: The conversation gets “difficult when someone isn’t open or can’t listen. […] I just want to get it out, but I don’t let any thoughts in” (EZF_5_ 00:17:01-00:17:22).  Other guests expressed similar thoughts: they describe the philosophical conversation as a movement in which “individual situations” are discussed “on a much larger level" (EZF_1_ 00:01:33), and for this, one must “Tak[e] off the 'psychology glasses'” (EZF_1_ 00:01:06). It is further stated: “The strength of philosophical counselling is that it’s not just personal” (EZF_7_ 00:34:21), it works on both levels, meta and personal. Philosophical counselling is indeed perceived as theoretical—but not entirely detached; rather, it maintains a connection to emotions or one's own assumptions: “So it’s both. Yes, and then that only really becomes meaningful, actually. So the theory only becomes meaningful by actually going through it.” EZF_6_00:12:19-00:12:29). |
| **1** | Questioning and philosophical texts | The philosophical way of questioning goes beyond everyday inquiry:  "[They] essentially questioned these constructs [of my thinking]. Why, how it even comes about that I think exactly this way [...] That’s what makes [Philosophical Practice] so unique, because it questions things one step further than what you’re usually accustomed to thinking about on these topics" (EZF_4_00:07:31 – 00:07:56). |
| **1** | Differences from psychotherapy | Another interviewee described the difference in nuanced terms. Although psychotherapy may not be judgmental in the usual sense, the search for causes—why someone thinks or feels a certain way—can still feel like a form of evaluation. Philosophical counselling, in contrast, was experienced as offering even more freedom of thought:  "I’ve also done psychotherapy, talk therapy, and group therapy. And I find a very clear difference with Philosophical Practice—what I’ve always liked so much about it: nothing is judged. There’s no right or wrong. I think that’s a very significant difference. I mean, I did that talk therapy, and I thought it was good, and I didn’t feel like I was being put into boxes or anything, but there’s still more of an emphasis on why things are the way they are and whether it could be that... [there’s] also more of a directive behind it. And I don’t find that in Philosophical Practice, at least from my experience. I find it very free. Just saying whatever comes to mind [...] without immediately being questioned, like, why are you thinking that now [...] I think that judgment factor isn’t there, and you don’t have to think, oh, can I even say this, or am I giving something away about myself. That’s what I find so wonderful about Philosophical Practice" (EZF_5_00:17:50-00:19:21). |
| **2** | Benevolent and intellectually stimulating | If necessary, the practitioner would prioritize the empathic, dialogical relationship over a shift to the meta-level or more critical questioning:  “When I was feeling overwhelmed by the topic […], the practitioner went along with me […], held me with their words, and let the topic go” (EZF_2_00:38:28-00:28:37). |
| **2** | Able to navigate the universe of philosophy and communicate as a human being on equal footing | "Guests, for example, appreciate the 'immense wealth of quotes, knowledge, simply all the things she has already read” (EZF_6_00:35:41 – 00:36:53), at the same time they experienced a Philosophical Practitioner as someone who could communicate at any level" (EZF_8_00:2:23).  One guest even described the practitioner as a kind of fellow seeker—and the dialogue itself as searching together:  “It’s one of the most gratifying things for me—finding, as a seeker, someone else who’s also seeking, and being able to mutually inspire each other” (EZF_7_00:03:31). |
| **2** | Listens deeply and contributes thoughtfully | In the following quote, for example, the practitioner stays close to the guest’s words, but highlights terms in a way that opens reflection:  "I particularly noticed this in situations where other people wouldn’t react or perhaps wouldn’t pick up on certain things. It was precisely those statements, which sometimes seemed so trivial to me, that she would pick up on and ask further questions about. And I thought that was really cool because it made me pause and wonder, “Oh, did I say something wrong?”—prompting me to reflect on myself. I really appreciated how she found a way to do this in a very kind manner, while still opening my eyes in the process" (EZF_4_00:21:34 - 00:22:12). |
| **3** | Lingering Resonance |  |
| **3** | Seeing differently | “Each time, the conversation showed me a different perspective on what I had brought in. It was really exciting to zoom out from the whole thing and look at it—maybe from above, but also from the side […]. I found it an incredibly valuable experience to really look at it philosophically as well […], and I always left the conversations feeling very enriched” (EZF_4_00:00:50–00:01:29).  This broadening of perspective becomes especially intense—and challenging—when it involves questioning one’s own assumptions. Another participant described the fruitful discomfort that can come with such philosophical questioning:  "What stuck with me most [...] was whether my assumptions were even valid. I mean, it was uncomfortable for me [...] but uncomfortable isn’t always bad. It can lead to something. It was an uncomfortable thought […] a kind of unsettling moment that helps you see things differently [...] Maybe it’s a seed that hasn’t fully surfaced yet. But it might" (EZF_2_00:24:23 – 00:26:37). |
| **3** | Other practical impacts |  |
| **3** | Stepping out |  |
| **3** | Inspiration | “We dared to really look at our own finitude – that was the *game-changer*, only in the philosophical conversation did that become *precious* to me. So even just that question. And the question—and my *very* *surprising*, to me surprising, answer” (EZF_1_ 00:04:50-00:05:12). |
| **3** | Consoling connectedness |  |
| **3** | **Philosophical curiosity as a stance toward life** | Being open to inquiry is seen as key: "I think what people need to bring to Philosophical Practice is curiosity, and a willingness not to expect answers but maybe even to leave with more questions than they came with [...] I find that enriching" (EZF_3_00:13:07–00:13:39). Philosophical inquiry here is not a means to an end, but an activity of the mind with value in and of itself. |
| **4** | Foundational attitude and acute psychological distress | "I think there is a boundary [...] I had the feeling that I already needed to be somewhat grounded to know where I stand, so that I could drift and still find my way back—not get lost along the way. Because [...] it does, in part, call your whole self into question. And if you're already lost, then there's nothing left to hold you" (EZF_4_00:29:47 – 00:30:11). |
| **4** | Manipulative philosophical counselling | "What concerns me—and this would also be a criticism—is when it's marketed like: 'Want to die well? Do philosophical counselling!' That's my worst nightmare—if it gets this self-optimisation vibe. You're dealing with this topic? Then Philosophical Practice will help you. After five sessions, you'll be completely at peace with your death. That’s when it flips. That mustn’t happen. And I've always told [the practitioner] that. [...] I think I'd even go so far as to say that it can also lead to people feeling more agitated." (EZF_3_00:30:30 – 00:30:24) |
| **4** | Philosophical counselling in societal contexts | “That is something important for the care sector, and it’s almost even more important in society to say that we really need many more spaces to talk about things without the aim of being right or proving someone right […] but rather to have a space to exchange: what do I understand, what do you understand, and what do we do with that now? I find that very moving as well, because I’ve realized that we simply need much more of this” (EZF_1_00:27:10-00:28:10).  “It’s not as if people who have a house and a boat say, ‘Wow, I’m so happy, I’m just floating through life, comfortably heading toward the end’—quite the opposite, really. And I can imagine that philosophical practice meets a need in our society, which is still very material […]. The question of meaning is being asked more and more. And people are more alone than before, because they determine themselves more. But then they also have more sorrow with that […]. This isn’t a problem you heal; the question is simply there. It isn’t an illness; it’s really a question of life: How do we want to live?” (EZF_8_00:22:55–00:24:10). |
