# Supplementary material for: ‘Stay Curious’: guest experiences of philosophical counselling in palliative care – a qualitative study
Source: BMC Palliat Care. 2026 Jun 29;25:271. doi: 10.1186/s12904-026-02178-x (PMC13576289; doi:10.1186/s12904-026-02178-x)
Supplement: Supplementary file 4 [file 12904_2026_2178_MOESM3_ESM.docx]

**Additional File 5 - Reflexivity and trustworthiness**

Author 1 (m). PSch (Dr., MPH, nurse), 12 years of experience in qualitative research, participatory research, and teaching in the field of palliative care, 8 years experience in nursing, 10 years experience in philosophical practice

Author 2 (f). SR (Dr., Mag., teacher, academic philosophical practitioner), 6 years of experience in qualitative participatory research, 4 years university teaching linguistics, multilingualism, and philosophical practice; 5 years school teaching and as volunteer in migration context.

Author 3 (f). SVR (MA, academic philosophical practitioner, systemic counsellor, certified social worker), 3 years of experience in qualitative research, 5 years of experience in philosophical practice, 10 years experience in social work.

Author 4 (m). KW (Dr., MA, sociologist and care researcher), 20 years experience in qualitative research, participatory research, and teaching in the field of hospice work, public health & end-of-life care, 10 years experience in the field of care research

Additional comments to:

COREQ Item 7-8

Participants received an information sheet. At the start of each interview, the interviewer introduced themself and their role within the conversational flow (dialogical introduction rather than checklist-style briefing), outlining background and study aims. No prior relationship existed beyond recruitment.

COREQ Item 23

Transcripts were not returned to participants for comment or correction; however, findings were discussed in a workshop with the participating philosophical practitioners to assess plausibility, and converged with results from other sub‑studies of the larger research project.
